# Case report: Advanced age at transplantation and pre-emptive treatment with dupilumab in DOCK8 deficiency

**DOI:** 10.3389/fimmu.2024.1507494

**Published:** 2025-01-28

**Authors:** Sophia Trombello, Andrea Jarisch, Andre Willasch, Eva Rettinger, Julia Fekadu-Siebald, Dirk Holzinger, Roland Adelmann, Peter Bader, Shahrzad Bakhtiar

**Affiliations:** ^1^ Division for Stem Cell Transplantation and Immunology, Department for Pediatrics, Goethe University Hospital Frankfurt, Frankfurt am Main, Germany; ^2^ Children’s Hospital, Heidelberg University Hospital, Heidelberg, Germany; ^3^ Department of Pediatric Hematology-Oncology, University of Duisburg-Essen, Essen, Germany; ^4^ Department of Applied Health Sciences, University of Applied Sciences Bochum, Bochum, Germany; ^5^ Department for Children and Adolescents Medicine, Hospital Oberberg, Gummersbach, Germany

**Keywords:** DOCK8-deficiency, alloHSCT, dupilumab, combined immunodeficiency, omalizumab

## Abstract

Dedicator of cytokinesis 8 (DOCK8) deficiency is a combined immunodeficiency (CID) due to biallelic mutations in the gene encoding DOCK8. Major clinical phenomena are recurrent severe infections of the lungs and skin, atopic eczema, and predisposition to malignancy leading to a poor prognosis. Typical findings include highly elevated IgE and eosinophilia. Allogeneic hematopoietic stem cell transplantation (alloHSCT) is indicated as the only curative treatment option. We present a patient with advanced disease undergoing alloHSCT at the age of 11 years after individualized pre-treatment using dupilumab and rituximab resulting in a decrease in IgE levels and clinical improvement of the skin condition. Additionally, in a review of the literature, we summarize morbidity and outcome in DOCK8-deficient patients older than 8 years of age receiving alloHSCT. Life-threatening infections, malignancy, and disease-related complications with organ damage pre-transplant are challenging in older DOCK8-deficient patients. The therapeutic role of dupilumab in DOCK8 deficiency should be evaluated in larger studies.

## 1 Introduction

Dedicator of cytokinesis 8 deficiency (DOCK8, OMIM 611432) is a combined immunodeficiency (1–3) with clinical presentation of severe susceptibility to infections, immune dysregulation as atopic disease, autoimmunity, and elevated IgE, as well as predisposition for cancer (4, 5). Atopic disease most commonly manifests as eczema and food allergies (5, 6). Most patients display severe viral infections of the skin due to herpes simplex virus (HSV), human papilloma virus (HPV), and molluscum contagiosum (MC), followed by bacterial skin abscesses and mucocutaneous candidiasis (5, 7). Rare features include vasculopathy in part associated with cerebral events and sclerosing cholangitis due to chronic infection with cryptosporidium (4, 5, 7, 8). Nearly all patients display highly elevated IgE. Increased levels of IgG and IgA, along with low levels of IgM, are seen in many cases (5). Chronic Epstein-Barr virus (EBV) viremia likely results in higher risk for malignancies (5, 9). DOCK8 protein (190 kDa, 1,701 amino acids, cytogenic localization 9p24.3) (10) is part of the actin remodeling process (11). Biallelic homozygous or compound heterozygous mutations or deletions in the DOCK8 gene impact the persistence and activation of CD8+ T and NK cells, dysfunctional regulatory T cells (Treg) (12), and cause an imbalanced differentiation and cytokine production of T helper cells (5, 8, 13–15). The differentiation of B cells and their capacity of immunoglobulin production are impaired as well (4, 13, 16–19). AlloHSCT is the only life-saving treatment in DOCK8-deficient patients (13, 20–22). The importance of an early alloHSCT and adequate control of disease complications pre-transplant has been shown (23, 24).

Aydin et al., on behalf of the EBMT Inborn Errors Working Party (IEWP), studied the natural course of the disease in DOCK8 deficiency. In their manuscript published in 2015, they showed that both overall survival and event-free survival rapidly decreased beyond the age of 10 years (4).

In 2019, a study was published on the outcome of alloHSCT in patients with DOCK8 deficiency, including data analysis by age at transplantation. Although the analysis did not reach significance level, there was a tendency toward inferior outcome for patients above the age of 8 years (22). Based on these two large EBMT IEWP studies, we decided to conduct a literature review and focus on patients over 8 years of age.

Literature search for alloHSCT in DOCK8 deficiency in patients older than 8 years included information on comorbidities, immune modulating treatment, post-transplantation complications, survival, and outcome. This retrospective study was performed in the Division for Stem Cell Transplantation and Immunology in Frankfurt/Main, Germany (EBMT Centre Code 138). Written informed consent was obtained from the parents. The study was approved by the local ethics committee of the Frankfurt Goethe University (IRB approval no. 167/16). The results of this study add valuable information to the literature, especially when clinicians counsel patients with a delayed diagnosis of DOCK8 deficiency.

## 2 Case description

**Figure 1:**
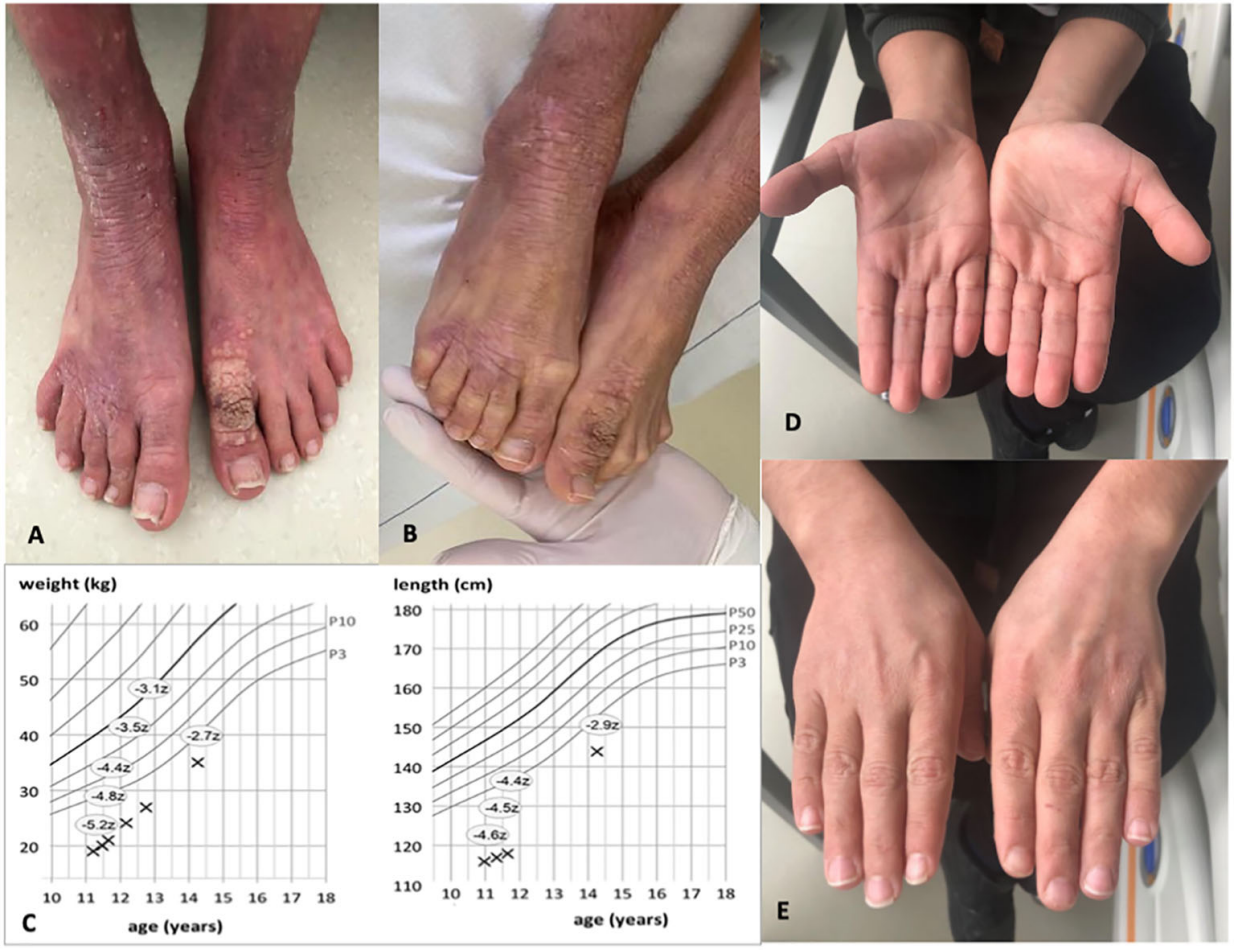
Images of the patient before and 1 year after alloHSCT. **(A, B)** Show patient’s feet with xerosis cutis, lichenification, and warts pre-alloHSCT. **(C)** indicates weight and length gain after alloHSCT. Z-scores measure the distance from the 50th percentile via standard deviation. **(D, E)** Show healthy skin of both hands with resolution of eczema post-alloHSCT.

**Figure 2:**
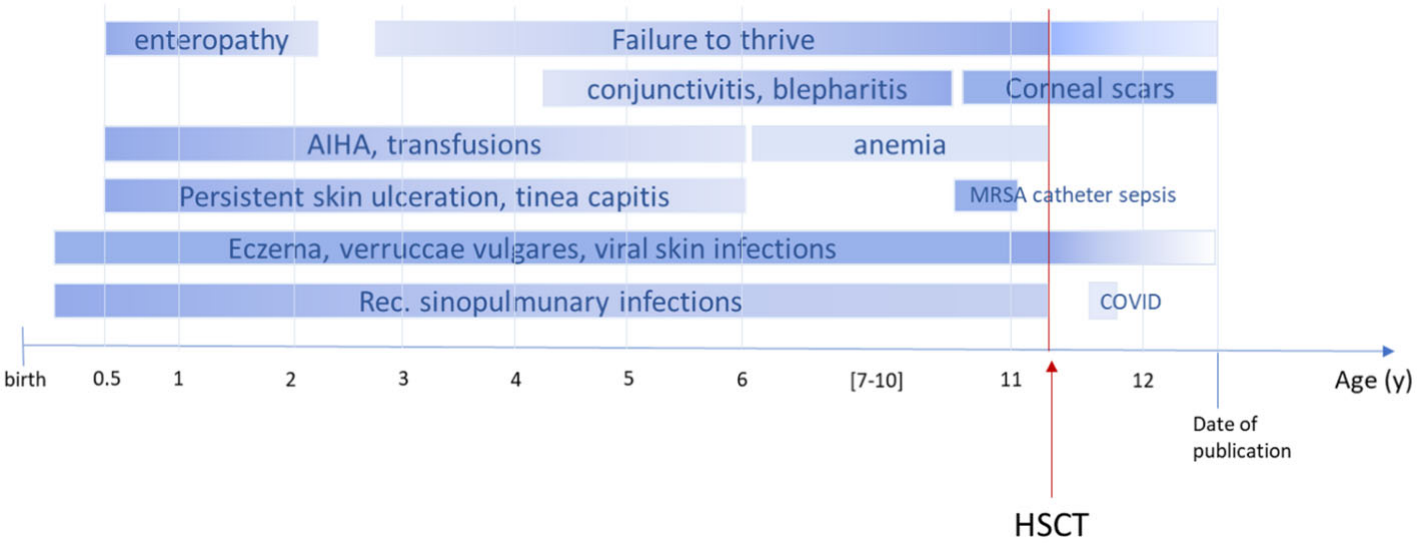
Timeline course of disease and symptoms pre- and post-alloHSCT. The figure illustrates the onset of symptoms in our patient. AIHA, autoimmune hemolytic anemia; MRSA, methicillin-resistant *Staphylococcus aureus*; COVID, coronavirus disease; shading indicates progress or regression; dark color refers to severity.

An 11-year-old boy of Syrian descent was referred for further treatment after a diagnosis of homozygous biallelic frameshift mutation in the DOCK8 gene (c.3339delT) was established. The patient suffered from inflammatory bowel disease, eczema, and transfusion-dependent autoimmune-hemolytic anemia since his early childhood. Skin diseases included verrucae vulgares due to HPV infection, tinea capitis, xerosis cutis, candida albicans, and superinfection with methicillin-resistant *Staphylococcus aureus* (MRSA) resulting in therapy-resistant ulcerations (**Figure 1**). Furthermore, he suffered from recurrent sinopulmonary infections and one episode of severe catheter-related sepsis due to MRSA. Ophthalmology work up revealed chronic conjunctivitis and uveitis, possibly due to the underlying CMV infection, resulting in corneal scars and loss of sight on both eyes. Evolving symptoms are displayed in **Figure 2**. He showed significant growth delay (**Figure 1**).

### 2.1 Immunological work up

Naïve CD4 (CD4+CD45RA+CD62L+) and CD8 (CD8+CD45RA+CD62L+) cells as well as terminally differentiated effector memory T cells re-expressing CD45RA (TEMRA) counts were reduced, while activated CD4+ and CD8+ (HLA-DR+) cells were increased. Regulatory T cells were within the normal range. CD19+ B-cell maturation was impaired with higher naïve B cells and decreased number of non-switched und switched memory B cells. Further findings included high levels of immunoglobulins (Ig): IgG of 2167 mg/dl (reference 700–1550 mg/dl), IgA of 582 mg/dl (reference 58–290 mg/dl), IgE of 34000 U/ml (reference <200 U/ml), and low IgM of 8 mg/dl (reference 49–180 mg/dl). Eosinophils were elevated at 2,000/µl (reference 20–700/µl) (detailed laboratory work up is shown in **Supplementary Table S1**).

### 2.2 Pre-treatment

**Figure 3:**
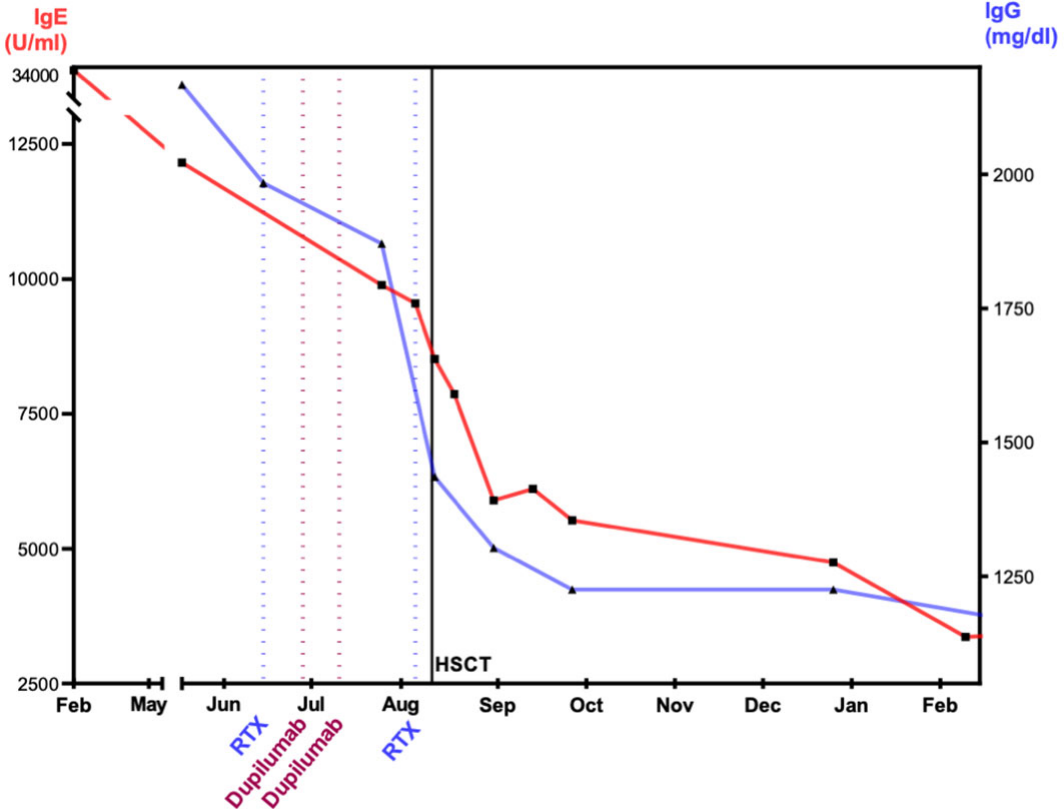
Effect of dupilumab, rituximab, and alloHSCT on IgE and IgG levels. The figure visualizes the level of immunoglobulins after immune-modulating therapy with dupilumab and rituximab. IgE, immunoglobulin E; IgG, immunoglobulin G; RTX, rituximab; alloHSCT, hematopoietic stem cell transplantation. IgE and IgG display decrease prior and after alloHSCT. At 1 year after alloHSCT, IgG shows normal levels. IgE decreased at 1 year and within the normal range at 2 years post-alloHSCT.

To reduce pre-existing inflammation and minimize the risk for inflammatory complications post-transplant, a treatment with dupilumab and rituximab was started. Dupilumab is a human monoclonal antibody (immunoglobulin G4 subclass) that suppresses the response to the cytokines IL-4 and IL-13 (25, 26) by blocking the shared subunit of IL-4 and IL-13 receptor (27). Dupilumab was administered subcutaneously bi-weekly with an initial loading dose of 2 × 300 mg and following dose of 300 mg. We observed a rapid decrease in IgE (**Figure 3**). Rituximab was administered twice to eliminate B cells as reservoir for EBV. The effect of rituximab is shown through the course of IgG levels (**Figure 3**). During alloHSCT and post-transplant, all investigations of EBV viremia remained negative.

### 2.3 AlloHSCT and outcome

The conditioning regimen consisted of treosulfan (12 g/m^2^/day), fludarabine (40 mg/m^2^), and thiotepa (5 mg/kg). For prophylaxis of graft-versus-host disease (GvHD) anti-thymocyte globulin (ATG) (20 mg/kg) and methotrexate (10 mg/m^2^ on days +2, +4, and +6) were used. A 10/10 HLA-matched unrelated donor (MUD) was available, from whom unmanipulated bone marrow was harvested. Cell dose was 6.19 × 10^6^ cells/kg of CD34+ and 75.3 × 10^6^/kg of CD3+ cells. Antiviral and antimycotic prophylaxis included acyclovir and fluconazole, followed by liposomal amphotericin B. The pre-treatment and the conditioning regimen were tolerated well without any signs of relevant organ toxicity. One episode of fever during aplasia required antibiotic treatment. Leukocyte and neutrophile recovery (cell count >500/µl) was achieved at day +17, thrombocyte recovery (cell count >50/nl) at day +25, and full donor chimerism was detected at day +28 and in all following assessments (bone marrow and peripheral blood). Starting with pre-transplant high count, T cells never fell below 1,000/µl. After the administration of rituximab, B cell reconstitution was delayed at day +90, which required transient intravenous substitution of immunoglobulins. No signs of acute or chronic GvHD appeared. Four months post-transplant, the patient presented with mild upper respiratory infection due to SARS-CoV-2 resulting in positive PCR through nasopharyngeal swab. We observed a full recovery from SARS-CoV-2 infection without additional antiviral or antibody treatment.

At the last follow up 2 years post-alloHSCT, the patient is alive and well without immunosuppression. The skin pathology is in complete remission, without evidence for eczema, warts, and/or GvHD. IgE level is within the normal range (150 U/ml) as well as eosinophile count, IgG, and IgA. We observed a rapid catch-up growth as shown in the course of growth and weight percentiles. He caught up weight from −5.2 to −2.7 standard deviations from the 50th percentile and length from −4.6 to −2.9 (**Figure 1**).

## 3 Review of the literature

The review of literature included a systemic research of Medline database from the National Library of Medicine (NLM) via PubMed using terms such as DOCK8, HSCT, dupilumab, and rituximab. Results were filtered for DOCK8-deficient patients who underwent alloHSCT at the age of 8 years or older. Duplications were excluded (13, 20, 28). Diagnosis in the screened references was determined through either genetic mutation detection and/or flow cytometrical detection of the loss of DOCK8 protein. Donors are considered matched [either matched sibling donors (MSD), or matched unknown donors (MUD)] if displaying an HLA-match of at least 9 out of 10.

**Figure 4:**
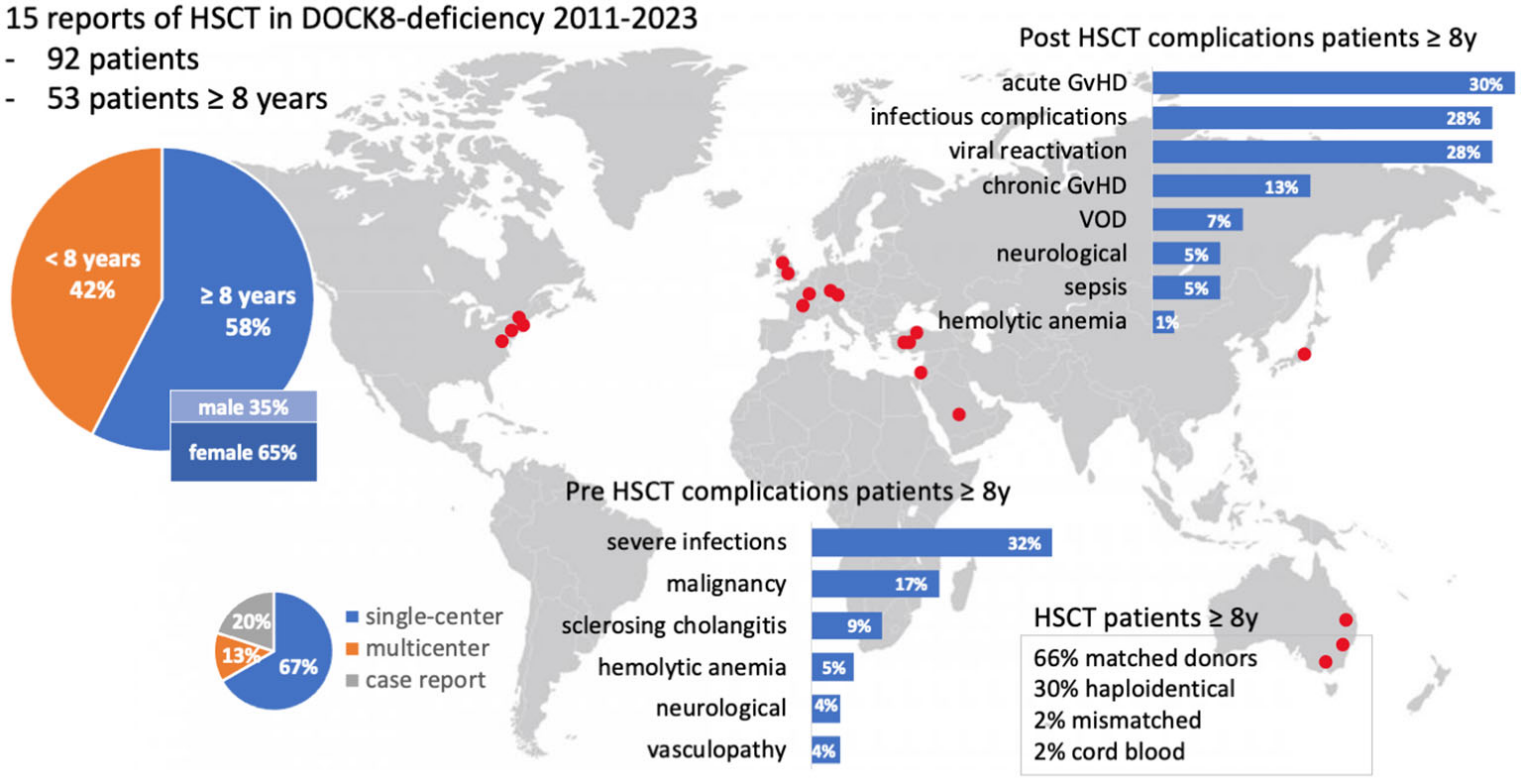
Visual abstract of the review of the literature. Fifteen publications on DOCK8 deficiency treated with alloHSCT were available, including single- and multicenter studies. Red dots indicate the locations of participating centers. A focus lies on patients with at least 8 years of age. Two bar charts illustrate pre- and post-HSCT complications (%). GvHD, graft-versus-host-disease; HSCT, hematopoietic stem cell transplantation; VOD, veno-occlusive disease; y, year.

The literature review revealed 173 patients with DOCK8 deficiency who underwent alloHSCT (**Figure 4**). Eighty-one out of 173 were reported and analyzed in a large study of Aydin et al. and will be referred to separately (22). Out of the remaining 92 patients, 53 were transplanted at the age of 8 years or older (53/92, 57.6%) (13, 20, 21, 23, 24, 28–37). Within this cohort of older patients, there were more female patients (64%; 34 females, 18 males). The age ranged from 8 to 27 years. Pre-transplant morbidity ranged from recurrent respiratory tract and skin infections with eczema in nearly all patients to less frequent complications as liver disease due to chronic cryptosporidium infection, vasculopathy, and malignancy. IgE levels ranged from 8.3 to 36.000 U/ml, while five cases were over 15.000 U/ml (12.5%) and two over 34.000 U/ml (5%). Immune-modulating pre-treatment was initiated in EBV-positive patients using rituximab (n = 6), and one patient was treated with mycophenolate mofetil (MMF) due to aortitis (35) (**Table 1**).

**Table 1:** Review of the literature part 1 (authors, publication year, type of study, transplantation year, number of patients with HSCT, number of patients ≥8 years at transplantation, sex distribution, concentration of IgE, anti-inflammatory pre-treatment, complications, and comorbidity prior to HSCT).

Report no.	Author	Year	Type	HSCT year	HSCT all	HSCT >8y (n)	Age at HSCT	Sex f:m	Range IgE (U/ml)	Anti-inflammatory drugs (n)	Pre-HSCT comorbidity other than atopic dermatitis, allergy or recurrent infections (n out of all >8y)
R1	Gatz et al.	2011	Retrospective, single center	appr. 2007-2009	2	2	10, 17	1:1	9196; 19400	RTX for chron. hemol.anemia (n=1)	2/2 rec. pneumonias, 1/2 chronic hemolytic anemia, 1/2 severe periodontitis
R2	**Barlogis** et al.	2011	Case report	appr. 2007-2009	2	2	9, 9	2:0	36000	RTX for EBV(n=1)	1/2 Vasculopathy celiac artery, EBV-prolif. synd.; 1/2 (**P1**) severe lung disease and chronic ulcerative herpes simplex virus infection
R3	**Al-Mousa** et al.	2013	Case report	appr. 2011	1	1	8	0:1	11000**	0	EBV, CMV chronic infection, BE, cryptosporidium infection with SC, severe diarrhea and dehydratation, sepsis, ITC-unit before HSCT (**P2**)
R4	Pai et al.	2014	Prospective	n.a.	6	1	10	1:0	n.a.	0	n.a.
R5	Cuellar-Rodriguez J et al.	2015	Prospective, single center	2012-2013	6	6	10, 16, 18, 23, 25, 27	4:2	8.3 – 11279***	0	6/6 rec. pneumonias, 4/6 restrictive ventilatory defects, 3/6 BE, 2/6 severe eczema, 1/6 hearing loss, 1/6 partial vision loss
R6	**Al-Herz** et al.	2016	Retrospective, two centers	appr. 2012-2015	11	4	8, 8, 10, 11	3:1	n.a.	RTX (n=2), etanercept for idiop. pneum.(n=1)	1/2 leiomyosarcoma, MRSA pericarditis, chron. cryptosporidium and Giardia infection; 1/2 living donor liver transplantation due to liver failure from cryptosporidium induced SC, appr. 72 days before HSCT
R7	**Shah** et al.	2017	Prospective, single center	2013-2015	7	6	10, 18, 19, 20, 20, 25	3:3	639-5970	RTX for Burkitt lymphoma (n=1)	3/4 malignancy (Burkitt, HL(**P4**), vulvar SCC), 1/4 stroke, 2/4 vasculopathy (renal art.stenosis, cardiomyop., ectatic thoracic aorta), 2/4 BE, 1/4 (**P4**) chron.liver disease + pulm. fibrosis + recurrent pancreatitis
R8	Uygun et al.	2017	Retrospective, single center	2013-2015	5	4	11, 11, 12, 13	4:0	240-10900	0	1/4 AIHA 4/4 pneumonia
R9	Kuskonmaz et al.	2018	Retrospective, single center	appr. 2011-2013	3	1	15	n.a.	2600	0	SC, giardiasis
R10	Shah et al./ **Freeman** et al.	2019	Addendum to Shah 2017	2013-2015	1	1	11	1:0	2331	0	end-stage liver disease, tandem living donor liver transplantation and HSCT, same donor, BE, multiple infectious complications, liver failure, VOD, graft failure (**P5**)
R11	Pillay et al.	2019	retrospective/ prospective multi-center	appr. 2016-2018	20	12*	19, 20, 20, 9, 18, 25, 19, 27, 10, 16, 16, 11*	7:5	38,5-10000	RTX (n=1)	1/4 EBV-lymphoma, 1/4 SCC, 1/4 vulvar SCC, 1/4 BE
R12	Haskologlu et al.	2020	Retrospective, single center	appr. 2012-2018	11	4	14, 15, 8, 9	2:2	358-36000	0/n.a.	3/4 hepatic fibrosis, 2/4 malignancy (cervical lymph node/ supraclavicular plasmocytoma), 2/4 IBD, celiac disease, 1/4 refractory ITP (splenectomy), 1/4 ITP and AIH, 1/4 AIT, 1/4 mastoiditis, 4/4 BE
R13	Ollech et al.	2021	Retrospective, single center	appr. 2020	6	1	12	0:1	n.a.	MMF (aortitis)	Aortitis, tinea corporis and capitis
R14	Raedler et al.	2021	Retrospective, single center	2004-2017	9	6	8, 9, 12, 13, 16, 17	5:1	median 17438	0	n.a.
R15	Kono et al.	2023	Retrospective single center	n.a.	2	2	16,22	1:1	n.a.	0	Acute eosinophilic pneumonia, cutaneous squamous cell carcinoma

Appr., approximately; RTX, rituximab; chron., chronic; hemol., hemolytic; idiop., idiopathic; rec., recurrent; ITC, intensive care; SC, sclerosing cholangitis; BE, bronchiectasis; idiop., idiopathic; pneum., pneumonia; MRSA, methicillin-resistant *Staphylococcus aureus*; art., artery; cardiomyop., cardiomyopathy; pulm., pulmonary; HL, Hodgkin lymphoma; SCC, squamous cell carcinoma; IBD, inflammatory bowel disease; ITP, immune thrombocytopenia; AIH, autoimmune hepatitis; AIT, autoimmune thyroiditis; HSCT, hematopoietic stem cell transplantation; TBI, total body irradiation; cGy, centigray; MMF, mycophenolate mofetil.

*A total of 13 patients were transplanted >8 years, but one is also reported by Cuellar-Rodriguez et al.; hence, we count 12.

**This patient before HSCT at the age of 6 years showed an IgE of 28.000 U/ml.

***This patient with an IgE level of 11,279 U/ml is also reported by Pillay et al. Publications which report patients 1 to 5 are pointed out bold.

### 3.1 AlloHSCT and outcome in patients >8 years of age

The conditioning regimens were busulfan-based (n = 36), treosulfan-based (n = 11), and fludarabine/melphalan-based (n = 3). In one case (P2), unconditioned matched-sibling donor transplantation was conducted presumably as a rescue strategy due to severe intensive care-dependent sepsis (31). Thirty-five patients (66%) received their graft from an MUD, while 34% underwent either mismatched unrelated or haploidentical alloHSCT.

Within the cohort by Aydin et al., 6/81 (7%) had a haploidentical donor, and 62/81 (70%) received their graft from a matched donor. Bone marrow was the major source of stem cells, 37/53 (69%), while 9/53 (17%) received peripheral blood stem cells (PBSC), and two patients received cord blood. Full chimerism was achieved in 45 of 53 patients (84%), very similar to the larger cohort reported by Aydin et al. (22). Acute GvHD [skin grades I–III (n = 10), intestinal grades I–III (n = 7), lung grade II (n = 1); no GvHD data available (n = 2)] occurred in 14 out of 53 patients (26%). Five out of 53 patients (9%) displayed chronic GvHD [gut (n = 1), skin (n = 2), myositis (n = 1), oral lichen planus (n = 1), mucosa (n = 1), liver (n = 1)] and one with suspected bronchiolitis obliterans syndrome (BOS). Acute GvHD occurred in 33% (27% grades II–IV and 11% grades III–IV) and chronic GvHD in 10% (three mild, two moderate, two severe) of patients.

Five patients (9%, P1-5) died within a range of +40 days (P5) to 1 year (P2) post-transplant (23, 28, 30, 31, 34). P1 died due to “transplant-related” cause after haploidentical alloHSCT, approximately in 2007, without further detailed information available (30). One patient (P2) underwent unconditioned alloHSCT of an MSD following treatment in an intensive care unit with sepsis and severe diarrhea, and dehydration due to cryptosporidium infection. Transplantation led to mixed chimerism. Death, 1 year after transplantation, was attributed to uncontrolled chronic GvHD (skin, gut) and sepsis (31). P3, despite MSD and full chimerism, deceased on day +58 due to an invasive *Klebsiella* species infection at the age of 8.9 years (23). A multifactorial reason for death was reported in P4 (at 25 years), including a history of Hodgkin’s lymphoma, subsequent bleomycin-associated pulmonary fibrosis, recurrent pneumonias, recurrent pancreatitis, and chronic cholestatic liver disease, aggravated by non-compliance, e.g., tobacco abuse (28). One patient (P5) died on day +40 after haploidentical alloHSCT, which had been conducted on day +70 after a living-donor liver transplantation from the same donor. Severe infections—including candida sepsis, acyclovir-resistant herpes simplex viremia, and resistant pseudomonas infection—complicated the course of alloHSCT. Further deterioration of the clinical condition occurred due to veno-occlusive disease (VOD), graft failure, and ultimately multi-organ failure (28, 34) (**Table 2**). Furthermore, 10 patients were reported to have suffered from a malignancy, 9 of whom survived and recovered fully by their last follow-up.

**Table 2:** Review of the literature part 2 (conditioning regimen, donor characteristics, chimerism, graft-versus-host-disease (GvHD), overall survival, death, reason for death, and post-HSCT complications other than GvHD out of all patients over 8 years of age at transplantation).

	Conditioning	Donor	Source	Chimerism	Acute GvHD	Chron. GvHD	OS (FU time range)	Death, reason	Complications post-HSCT other than GvHD
R1	Flu+Melph+ATG + BM-targ. radiolabled MoAbs at 16 Gy BM dose (n=2)	MUD	BM, PB	full	0	0	2/2 (19mon-4y)		1/2 severe oral mucositis, cerebral abscesses (full recovery), EBV-reactivation; 2/2 flaring-up MC
R2	Bu+Cy(n=1); n.a.(n=1)	Haplo(**P1**) MSD (n=1)	BM	n.a. (**P1**), full(n=1)	(**P1** n.a.)	(**P1** n.a)	1/2 (24mon)	**P1**: 9y, transplant-related after haplo HSCT (appr. 2007)	
R3	Unconditioned (n=1)	MSD	BM	mixed	n=1	n=1	0	**P2**: 8y, uncontrollable cGvHD, Sepsis, 1y post HSCT	Sepsis
R4	Bu+Cy (n=1)	MSD	n.a.	full	n.a.	n.a.	1/1 (n.a.)		n.a.
R5	Bu+Flu (n=6)	MRD (n=3) MSD (n=3)	4xBM 2xPB	full	n=2 (°II+III)	(suspected BOS n=1)	6/6 (14-35mon)		3/6 sinopulmonary infection flaring up well responsive to AB, 1/6 (lymphoma): extensive lung mass, left lung collapse, mucous plug
R6	Bu+Flu (n=2), Bu+Cy (**P3,** n=1), Bu+Flu+ATG (n=1)	MSD (n=4)	BM	full	0	0	3/4 (0.6y - 4,2y)	**P3**: 8.9y at day +58 Klebsiella species sepsis	1/4 CMV-reactivation, persistent and worsening CNS white matter changes (leukoencephalopathy), idiop. pneu. treated with etanercept; 1/4 neurol. symptoms of unclear cause, pericardial effusion, presumed viral menigoenc. responsive to foscarnet; 1/4 CMV pneumonitis, EBV viremia, transient pseudotumor cerebri; 1/4 (**P3**) Klebsiella sepsis
R7	Bu+Flu + PT/Cy + 200cGy TBI (n=6)	Haplo (n=6)	BM	full	n=4 (°I-°III)	0	5/6 (9.5 - 31,7mon)	**P4**: 25y, multifactorial: worsening pulmonary fibrosis, rec. pneumonia, tobacco abuse, day +165	transient worsening of sinopulmonary infection, viral reactivation
R8	Bu+Flu+ATG (n=4); NM (CB, n=1)	MUD	3xBM 1xCB	full (n=3), GF (n=1)	n=2 (°III)	0	4/4 (12-32mon)		1/4 graft rejection, cholelithiasis (CB), 3/4 CMV-reac, 2/4 PRES, 1/4 herpetic dermatitis, 1/4 osteomyelitis, aspergillosis, 1/4 catheter related thrombosis, genital herpetic lesions, transient pancytopenia
R9	Bu+Cy (n=1)	MSD	BM	full	0	0	1/1 (64mon)		VOD
R10	Bu+Flu + PT/Cy + TBI 200 cGy (n=1)	Haplo (n=1)	BM	n.a.	0	0	0	**P5**: 11y, VOD, GF, Candida sepsis, Aciclovir resistent HSV-viremia, day +40	VOD, graft failure, aciclovir resistent HSV infection, VOD associated multiorgan failure with Candida sepsis on day +40 (HSCT at d+70 after liver transplantation)
R11	Bu+Flu(n=10)+Cy(n=3, haplo), Treo+Flu+Thio+ATG(n=1, haplo), Treo+Flu+Cam(n=1)	Haplo (n=3) MUD (n=5) MRD (n=4)	9xBM 3xPB	89.3% (n=1); full (n=10)	n=3 (°II-°III)	n=2	12/12 (6-23 mon)		post-transplant hemolytic anemia
R12	Treo+Flu (n=2); Treo+Flu+ATG (n=1, MMUD); Bu+Flu (n=1)	MMUD (n=1) MSD (n=2) MRD (n=1)	3xBM 1xPB	full	n=2 (°II-°III)	n=2	4/4 (14-71mon)		2/4 CMV reactivation and VOD, 1/4 hemorrhagic cystitis, acute renal injury
R13	n.a.	MSD	n.a.	n.a.	n.a.	n.a.	full (1mon)		
R14	Treo+Flu+Alem	(n=2)MSD (n=3) MUD (n=1) MRD	1xPBSC 5xBM	full (n=4), 75-90% (n=2)	n=1 (°II)	0	full (33-102mon)		Cervical lymphadenopathy with peripheral facial palsy, 3/6 CMV, 3/6 BK-, JC-viremia, EBV, 4/6 HSV, rec. osteomyelitis, abscesses left arm, molluscum contagiosa
R15	Flu+Melph+TBI; Bu+Flu+TBI	Haplo (n=1), CB (n=1)	PBSC, CB	full (n=2)	n=1 (°I)	1 (mild skin)	full (1-6y)		2/2 Catheter-related infection, CMV reactivation

Flu, fludarabine; Melph, melphalan; ATG, anti-thymocyte globulin; BM, bone marrow; MoAbs, monoclonal antibodies; Gy, gray; Bu, busulfan; Cy, cyclophosphamide; PT/Cy, post-transplantation cyclophosphamide; haplo, haploidentical; NM, non myelo-ablative; CB, cord blood; Cam, Campath (Alemtuzumab); GF, graft failure; Treo, treosulfan; MMUD, mis-matched unrelated donor; MUD, matched unrelated donor; MSD, matched sibling donor; OS, overall survival; FU, follow up; HSCT, hematopoietic stem cell transplantation; cGvHD, chronic graft-versus-host-disease; y, year; mon, month; VOD, veno-occlusive disease; MC, molluscum contagiosum; EBV, Epstein–Barr virus; CMV, cytomegaly virus; CNS, central nervous system; meningoenc., meningoencephalitis; BOS, bronchiolitis obliterans syndrome. P, Patients; Patients 1 to 5 are pointed out bold.

## 4 Discussion

In this work we present an 11-year-old DOCK8-deficient patient with a severe inflammatory disease manifestation and very high IgE levels. To reduce the inflammatory disease prior to alloHSCT, dupilumab was used in combination with rituximab. A substantial decrease in IgE levels and amelioration of skin eczema were achieved. Treatment was well tolerated without adverse events.

Dupilumab, an IL-4/IL-13 receptor inhibitor, suppresses the overwhelming T-helper type 2 inflammatory response (13, 38–40). There is growing evidence for the use of dupilumab either bi-weekly or once every 4 weeks in patients with immunodeficiency syndromes (35, 41–45). The bi-weekly administration seems to be more effective (40). To our knowledge, there is no larger study available on pre-transplantation use of dupilumab in pediatric patients. Nevertheless, there is some evidence for the efficacy of dupilumab in DOCK8 deficiency (35, 45, 46). Ollech et al. described two patients (2 and 10 years of age) receiving dupilumab and discussed the use of dupilumab as a bridge to alloHSCT. However, at the time of publication, none of their patients had received an alloHSCT (35). Additionally, we could find one recent case report on dupilumab and DOCK8 deficiency with high IgE levels in a 6-year-old girl resulting in a successful remission of disseminated eczema herpeticum (46).

Our case is the first reported DOCK8-deficient patient who underwent alloHSCT shortly after treatment with dupilumab. One year post-transplant, the patient’s IgE levels remained slightly elevated. Prolonged elevation of IgE post-transplant in DOCK8 deficiency has been reported in the literature and presumably is due to long lasting chemo-evading plasma B cells, which decline with variable delay (22, 23, 47). The eosinophile count increased after initiation of treatment and eventually decreased over time. This phenomenon is also reported in the literature (25). The patient’s eczema improved significantly before alloHSCT and eventually disappeared within a few weeks after transplantation. No skin toxicity or skin GvHD occurred in our patient. This might be due to the effective control of the patient’s skin disease by pre-transplant use of dupilumab and should be investigated in a larger cohort of patients with DOCK8 deficiency undergoing alloHSCT.

In our patient omalizumab, which is an anti-IgE antibody, was discussed at the time of treatment initiation (2020). Since there is an IgE level and body weight-dependent dosing strategy recommended for this antibody, we decided not to utilize omlizumab for our patient.

The current dosing recommendation is given only for IgE levels below 1,300 IU/ml in the US and 1,500 IU/ml in EU (48). In a study by Menzella et al. published in 2023, the authors conducted a detailed literature review on the efficacy and safety of omalizumab in patients with severe allergic asthma and other allergic diseases focusing on data of patients with higher IgE levels (49). This literature review included a small number of patients with IgE levels above 1,500 IU/ml. Also, in these patients, a beneficial effect of omalizumab could be shown, while there were no severe adverse events reported. Therefore, the authors suggested further analyzing the optimal dose in patients with (very) high IgE levels and eventually extending the dosing recommendation for this patient group (49). Comparative data for the efficacy of dupilumab versus omalizumab for patients with (very) high IgE levels are not available; nevertheless, we postulate that dupilumab might be more effective in this setting as it targets the hyper-IgE disease at the level of cell signaling.

Patients affected by DOCK8 deficiency suffer from combined immune deficiency with severe immune dysregulation. Clinical presentation and laboratory findings might overlap with other IEI, including “treg-o-pathies” (16, 50, 51), Wiskott–Aldrich syndrome (WAS) (4, 52) and hyper-IgE syndromes (autosomal dominant and recessive HIES) (15, 53). Unlike HIES, there are usually no syndromic features in DOCK8 deficiency (4, 54). Patients’ long-term outcome is poor due to an accumulation of life-threatening infections, especially by the age of 20 years, and a predisposition to malignancies (4). To date, alloHSCT is the only curative treatment option (13, 20, 22, 24, 28, 52). Several cohorts have shown promising results also for patients receiving a haploidentical transplantation (28, 37, 55). In almost all surviving patients, the skin disease resolved after successful alloHSCT (13, 22, 24, 28). In a few cases, other comorbidities, including vasculopathy (28, 30, 35), stroke (28), and malignancy (23, 24, 28), were also reported as being in remission. In some cases, remaining food allergies were described (23, 47). There is common agreement on offering alloHSCT to the affected individuals at an early stage of the disease, prior to the accumulation of organ damage, to achieve an optimal transplantation outcome (20, 24, 28, 30, 36). Fatal outcome was observed in five patients (P1, P2, P3, P4, P5), including three patients receiving a haploidentical graft (P1, P4, P 5). For P1, data was incomplete. In P2, P4, and P5, there was a high disease activity pre-transplant. P3 suffered from fulminant bacterial infection at +d58 post-alloHSCT. With regard to malignancy in DOCK8 deficiency, the predisposition to virus-driven malignancy after an alloHSCT remains to be investigated. A successful alloHSCT results in an excellent CD3+-chimerism (13, 20, 24) and recovery of CD8+ cytotoxic T-cell response after alloHSCT (13), which should be preventive of viral-driven malignancy development; nevertheless, long-term data focusing on malignancy and late-onset post-transplant complications are indicated.

In this review of the literature, we focused on DOCK8-deficient patients over 8 years of age receiving alloHSCT and pointed out severe comorbidities and complications related to the accumulation of organ damage by the underlying disease. Especially, for patients with high disease activity, an anti-inflammatory pre-treatment prior to alloHSCT is indicated. A short course of treatment by dupilumab was beneficial in our patient. Further studies in larger IEI cohorts with elevated IgE levels are indicated to confirm a possible improvement of the transplant outcome in these patients.

## Data Availability

The raw data supporting the conclusions of this article will be made available by the authors, without undue reservation.

## Supplementary material

Supplementary Table 1 Laboratory work up of the patient with dedicator of cytokinesis 8 deficiency. WBC, white blood cell count; Hb, hemoglobin; NK, natural killer cells; DPT, double positive T cells; DNT, double-negative T cells; T4, CD4+ T helper cells; T8, CD8+ cytotoxic T cells; Ig, immunoglobulin; TSH, thyroid stimulation hormone; fT4, free thyroxine; fT3, free triiodothyronine; Ab, antibody; TG, thyroglobulin; TPO, thyroid peroxidase, TSHR, thyroid-stimulating hormone receptor; hGH, human growth hormone, IGFBP-3, insulin-like growth factor binding protein 3; IGF-1, insulin-like growth factor 1; LH, luteinizing hormone; FSH, follicle-stimulating hormone.

## References

[1] Zhang Y Yu X Ichikawa M Lyons JJ Datta S Lamborn IT . Autosomal recessive phosphoglucomutase 3 (PGM3) mutations link glycosylation defects to atopy, immune deficiency, autoimmunity, and neurocognitive impairment. J Allergy Clin Immunol. (2014) 133(5):1400–9. doi: 10.1016/j.jaci.2014.02.013 PMC4016982 24589341

[2] Engelhardt KR McGhee S Winkler S Sassi A Woellner C Lopez-Herrera G . Large deletions and point mutations involving DOCK8 in the autosomal recessive form of the hyper-IgE syndrome. J Allergy Clin Immunol. (2009) 124(6):1289. doi: 10.1016/j.jaci.2009.10.038 20004785 PMC2818862

[3] Randall KL Chan SSY Ma CS Fung I Mei Y Yabas M . DOCK8 deficiency impairs CD8 T cell survival and function in humans and mice. J Exp Med. (2011) 208(11):2305–20. doi: 10.1084/jem.20110345 PMC3201196 22006977

[4] Aydin SE Kilic SS Aytekin C Kumar A Porras O Kainulainen L . DOCK8 deficiency: clinical and immunological phenotype and treatment options - a review of 136 patients. J Clin Immunol. (2015) 35(2):189–98. doi: 10.1007/s10875-014-0126-0 25627830

[5] Engelhardt KR Gertz ME Keles S Schäffer AA Sigmund EC Glocker C . The extended clinical phenotype of 64 patients with dedicator of cytokinesis 8 deficiency. J Allergy Clin Immunol. (2015) 136(2):402–12. doi: 10.1016/j.jaci.2014.12.1945 PMC4530066 25724123

[6] Boos AC Hagl B Schlesinger A Halm BE Ballenberger N Pinarci M . Atopic dermatitis, STAT3- and DOCK8-hyper-IgE syndromes differ in IgE-based sensitization pattern. Allergy. (2014) 69(7):943–53. doi: 10.1111/all.12416 24898675

[7] Chu EY Freeman AF Jing H Cowen EW Davis J Su HC . Cutaneous manifestations of DOCK8 deficiency syndrome. Arch Dermatol. (2012) 148(1):79–84. doi: 10.1001/archdermatol.2011.262 21931011 PMC4103903

[8] Su HC Jing H Angelus P Freeman AF . Insights into immunity from clinical and basic science studies of DOCK8 immunodeficiency syndrome. Immunol Rev. (2019) 287(1):9–19. doi: 10.1111/imr.12723 30565250 PMC6350515

[9] Biggs CM Keles S Chatila TA . DOCK8 deficiency: Insights into pathophysiology, clinical features and management. Clin Immunol. (2017) 181:75–82. doi: 10.1016/j.clim.2017.06.003 28625885 PMC5555255

[10] Ruusala A Aspenström P . Isolation and characterisation of DOCK8, a member of the DOCK180-related regulators of cell morphology. FEBS Lett. (2004) 572(1–3):159–66. doi: 10.1016/j.febslet.2004.06.095 15304341

[11] McGhee SA Chatila TA . DOCK8 immune deficiency as a model for primary cytoskeletal dysfunction. Dis Markers. (2010) 29(3–4):151–6. doi: 10.1155/2010/397291 PMC3835630 21178274

[12] Janssen E Morbach H Ullas S Bannock JM Massad C Menard L . DOCK8 deficient patients have a breakdown in peripheral B cell tolerance and defective regulatory T cells. J Allergy Clin Immunol. (2014) 134(6):1365–74. doi: 10.1016/j.jaci.2014.07.042 PMC4261031 25218284

[13] Pillay BA Avery DT Smart JM Cole T Choo S Chan D . Hematopoietic stem cell transplant effectively rescues lymphocyte differentiation and function in DOCK8-deficient patients. JCI Insight. (2019) 5:e127527. doi: 10.1172/jci.insight.127527 31021819 PMC6629099

[14] Tangye SG Pillay B Randall KL Avery DT Phan TG Gray P . Dedicator of cytokinesis 8-deficient CD4+ T cells are biased to a TH2 effector fate at the expense of TH1 and TH17 cells. J Allergy Clin Immunol. (2017) 139(3):933–49. doi: 10.1016/j.jaci.2016.07.016 PMC10500883 27554822

[15] Keles S Charbonnier LM Kabaleeswaran V Reisli I Genel F Gulez N . DOCK8 regulates STAT3 activation and promotes Th17 cell differentiation. J Allergy Clin Immunol. (2016) 138(5):1384–1394.e2. doi: 10.1016/j.jaci.2016.04.023 27350570 PMC5099100

[16] Alroqi FJ Charbonnier LM Keles S Ghandour F Mouawad P Sabouneh R . DOCK8 deficiency presenting as an IPEX-like disorder. J Clin Immunol. (2017) 37(8):811–9. doi: 10.1007/s10875-017-0451-1 PMC5691358 29058101

[17] Randall KL Lambe T Johnson AL Johnson A Treanor B Kucharska E . Dock8 mutations cripple B cell immunological synapses, germinal centers and long-lived antibody production. Nat Immunol. (2009) 10(12):1283–91. doi: 10.1038/ni.1820 PMC3437189 19898472

[18] Jabara HH McDonald DR Janssen E Massaad MJ Ramesh N Borzutzky A . DOCK8 functions as an adaptor that links toll-like receptor–MyD88 signaling to B cell activation. Nat Immunol. (2012) 13(6):612–20. doi: 10.1038/ni.2305 PMC3362684 22581261

[19] Mizesko MC Banerjee PP Monaco-Shawver L Mace EM Bernal WE Sawalle-Belohradsky J . Defective actin accumulation impairs human natural killer cell function in patients with dedicator of cytokinesis 8 deficiency. J Allergy Clin Immunol. (2013) 131(3):840–8. doi: 10.1016/j.jaci.2012.12.1568 PMC3646579 23380217

[20] Cuellar-Rodriguez J Freeman AF Grossman J Su H Parta M Murdock H . Matched related and unrelated donor hematopoietic stem cell transplantation for DOCK8 deficiency. Biol Blood Marrow Transplant. (2015) 21(6):1037–45. doi: 10.1016/j.bbmt.2015.01.022 PMC4426076 25636378

[21] Kuşkonmaz B Ayvaz D Tezcan İ Yüce A Sanal Ö Çetinkaya DU . Successful hematopoietic stem cell transplantation after myeloablative conditioning in three patients with dedicator of cytokinesis 8 deficiency (DOCK8) related hyper IgE syndrome. Bone Marrow Transplant. (2018) 53(3):339–43. doi: 10.1038/s41409-017-0040-1 29269803

[22] Aydin SE Freeman AF Al-Herz W Al-Mousa HA Arnaout RK Aydin RC . Hematopoietic stem cell transplantation as treatment for patients with DOCK8 deficiency. J Allergy Clin Immunol Pract. (2019) 7(3):848–55. doi: 10.1016/j.jaip.2018.10.035 PMC6771433 30391550

[23] Al-Herz W Chu JI van der Spek J Raghupathy R Massaad MJ Keles S . Hematopoietic stem cell transplantation outcomes for 11 patients with dedicator of cytokinesis 8 deficiency. J Allergy Clin Immunol. (2016) 138(3):852–859.e3. doi: 10.1016/j.jaci.2016.02.022 27130861 PMC5354354

[24] Haskologlu S Kostel Bal S Islamoglu C Aytekin C Guner S Sevinc S . Clinical, immunological features and follow up of 20 patients with dedicator of cytokinesis 8 (DOCK8) deficiency. Pediatr Allergy Immunol. (2020) 31(5):515–27. doi: 10.1111/pai.13236 PMC7228270 32108967

[25] Castro M Corren J Pavord ID Maspero J Wenzel S Rabe KF . Dupilumab efficacy and safety in moderate-to-Severe uncontrolled asthma. N Engl J Med. (2018) 378(26):2486–96. doi: 10.1056/NEJMoa1804092 29782217

[26] Marone G Granata F Pucino V Pecoraro A Heffler E Loffredo S . The intriguing role of interleukin 13 in the pathophysiology of asthma. Front Pharmacol. (2019) 10:1387. doi: 10.3389/fphar.2019.01387 31866859 PMC6908970

[27] Beck LA Thaçi D Hamilton JD Graham NM Bieber T Rocklin R . Dupilumab treatment in adults with moderate-to-severe atopic dermatitis. N Engl J Med. (2014) 371(2):130–9. doi: 10.1056/NEJMoa1314768 25006719

[28] Shah NN Freeman AF Su H Cole K Parta M Moutsopoulos NM . Haploidentical related donor hematopoietic stem cell transplantation for dedicator-of-Cytokinesis 8 deficiency using post-transplantation cyclophosphamide. Biol Blood Marrow Transplant. (2017) 23(6):980–90. doi: 10.1016/j.bbmt.2017.03.016 PMC5757872 28288951

[29] Gatz SA Benninghoff U Schütz C Schulz A Hönig M Pannicke U . Curative treatment of autosomal-recessive hyper-IgE syndrome by hematopoietic cell transplantation. Bone Marrow Transplant. (2011) 46(4):552–6. 10.1038/bmt.2010.169 20622910

[30] Barlogis V Galambrun C Chambost H Lamoureux-Toth S Petit P Stephan JL . Successful allogeneic hematopoietic stem cell transplantation for DOCK8 deficiency. J Allergy Clin Immunol. (2011) 128(2):420–422.e2. doi: 10.1016/j.jaci.2011.03.025 21546070

[31] Al-Mousa H Hawwari A Alsum Z . In DOCK8 deficiency donor cell engraftment post-genoidentical hematopoietic stem cell transplantation is possible without conditioning. J Allergy Clin Immunol. (2013) 131(4):1244–5. 10.1016/j.jaci.2012.12.663 23352633

[32] Pai SY . Treatment of primary immunodeficiency with allogeneic transplant and gene therapy. Hematology. (2019) 2019(1):457–65. 10.1182/hematology.2019000052 PMC6913427 31808905

[33] Uygun DFK Uygun V Reisli İ Keleş S Özen A Yılmaz M . Hematopoietic stem cell transplantation from unrelated donors in children with DOCK8 deficiency. Pediatr Transplant. (2017) 21(7). 10.1111/petr.13015 28664550

[34] Shah NN Freeman AF Hickstein DD . Addendum to: Haploidentical Related Donor Hematopoietic Stem Cell Transplantation for DOCK8 Deficiency Using Post-Transplantation Cyclophosphamide. Biol Blood Marrow Transplant. (2019) 25(2):e65–7. 10.1016/j.bbmt.2018.11.014 PMC8335748 30472434

[35] Ollech A Mashiah J Lev A Simon AJ Somech R Adam E . Treatment options for DOCK8 deficiency-related severe dermatitis. J Dermatol. (2021) 48(9):1386–93. 10.1111/1346-8138.15955 34043252

[36] Raedler J Magg T Rohlfs M Klein C Vallee T Hauck F . Lineage-specific chimerism and outcome after hematopoietic stem cell transplantation for DOCK8 deficiency. J Clin Immunol. (2021) 41(7):1536–48. doi: 10.1007/s10875-021-01069-5 PMC8452590 34080085

[37] Kono A Wakamatsu M Umezawa Y Muramatsu H Fujiwara H Tomomasa D . Successful treatment of DOCK8 deficiency by allogeneic hematopoietic cell transplantation from alternative donors. Int J Hematol. (2023) 118(4):519–25. doi: 10.1007/s12185-023-03613-y 37131080

[38] LaPorte SL Juo ZS Vaclavikova J Colf LA Qi X Heller NM . Molecular and structural basis of cytokine receptor pleiotropy in the interleukin-4/13 system. Cell. (2008) 132(2):259–72. doi: 10.1016/j.cell.2007.12.030 PMC2265076 18243101

[39] Gandhi NA Pirozzi G Graham NMH . Commonality of the IL-4/IL-13 pathway in atopic diseases. Expert Rev Clin Immunol. (2017) 13(5):425–37. doi: 10.1080/1744666X.2017.1298443 28277826

[40] Simpson EL Bieber T Guttman-Yassky E Beck LA Blauvelt A Cork MJ . Two phase 3 trials of dupilumab versus placebo in atopic dermatitis. N Engl J Med. (2016) 375(24):2335–48. doi: 10.1056/NEJMoa1610020 27690741

[41] Lévy R Béziat V Barbieux C Puel A Bourrat E Casanova JL . Efficacy of dupilumab for controlling severe atopic dermatitis in a patient with hyper-IgE syndrome. J Clin Immunol. (2020) 40(2):418–20. doi: 10.1007/s10875-020-00751-4 PMC9642001 31993867

[42] Sogkas G Hirsch S Jablonka A Witte T Schmidt RE Atschekzei F . Dupilumab to treat severe atopic dermatitis in autosomal dominant hyper-IgE syndrome. Clin Immunol. (2020) 215:108452. doi: 10.1016/j.clim.2020.108452 32360519

[43] Votquenne N Dupire G Michel O Ben Said B . Dupilumab for severe generalized eczematous eruption complicating common variable immunodeficiency. Eur J Dermatol. (2021) 31(1):93–4. doi: 10.1684/ejd.2020.3954 33586652

[44] Charvet E Bourrat E Hickman G Donadieu J Bellanné-Chantelot C Jachiet M . Efficacy of dupilumab for controlling severe atopic dermatitis with dominant-negative CARD11 variant. Clin Exp Dermatol. (2021) 46(7):1334–5. doi: 10.1111/ced.14686 33864281

[45] Guo T Wei L Karki S Wen S Li Q Lin Y . Omalizumab and dupilumab for the treatment of autosomal-recessive DOCK8 hyper-IgE syndrome. Indian J Dermatol Venereol Leprol (2024) 1–3. doi: 10.25259/IJDVL_348_2023 38031685

[46] Johar RA Hasanain A Khouqeer Y . Efficacy of dupilumab in treating atopic dermatitis with recurrent eczema herpeticum in a patient with DOCK8-deficiency hyper-IgE syndrome: A case report. Cureus 15(8):e43360. doi: 10.7759/cureus.43360 37701007 PMC10494277

[47] Happel CS Stone KD Freeman AF Shah NN Wang A Lyons JJ . Food allergies can persist after myeloablative hematopoietic stem cell transplantation in DOCK8-deficient patients. J Allergy Clin Immunol. (2016) 137(6):1895–1898.e5. doi: 10.1016/j.jaci.2015.11.017 26827248 PMC4899149

[48] Xolair | european medicines agency (EMA) [Internet]. (2009). Available at: https://www.ema.europa.eu/en/medicines/human/EPAR/xolair (Accessed 2024 Nov 27).

[49] Menzella F Just J Sauerbeck IS Mailaender C Saccheri F Thonnelier C . Omalizumab for the treatment of patients with severe allergic asthma with immunoglobulin e levels above >1500 IU/mL. World Allergy Organ J. (2023) 16(6):100787. doi: 10.1016/j.waojou.2023.100787 37332525 PMC10276275

[50] Gambineri E Ciullini Mannurita S Hagin D Vignoli M Anover-Sombke S DeBoer S . Clinical, immunological, and molecular heterogeneity of 173 patients with the phenotype of immune dysregulation, polyendocrinopathy, enteropathy, x-linked (IPEX) syndrome. Front Immunol [Internet] 9. https://www.ncbi.nlm.nih.gov/pmc/articles/PMC6223101/. doi: 10.3389/fimmu.2018.02411 PMC6223101 30443250

[51] Singh AK Eken A Hagin D Komal K Bhise G Shaji A . DOCK8 regulates fitness and function of regulatory T cells through modulation of IL-2 signaling. JCI Insight. (2017) 2(19):e94275, 94275. doi: 10.1172/jci.insight.94275 28978795 PMC5841873

[52] Albert MH Freeman AF . Wiskott-aldrich syndrome (WAS) and dedicator of cytokinesis 8- (DOCK8) deficiency. Front Pediatr. (2019) 7:451. doi: 10.3389/fped.2019.00451 31750279 PMC6848221

[53] Béziat V Li J Lin JX Ma CS Li P Bousfiha A . A recessive form of hyper-IgE syndrome by disruption of ZNF341-dependent STAT3 transcription and activity. Sci Immunol. (2018) 3(24):eaat4956. doi: 10.3389/fped.2019.00451 29907691 PMC6141026

[54] Ma CS Tangye SG . Flow cytometric-based analysis of defects in lymphocyte differentiation and function due to inborn errors of immunity. Front Immunol [Internet] 10:2108/full. doi: 10.3389/fimmu.2019.02108/full PMC6737833 31552044

[55] Ghosh S Schuster FR Adams O Babor F Borkhardt A Comoli P . Haploidentical stem cell transplantation in DOCK8 deficiency - successful control of pre-existing severe viremia with a TCRaß/CD19-depleted graft and antiviral treatment. Clin Immunol. (2014) 152(1–2):111–4. doi: 10.1016/j.clim.2014.03.006 24667686

