# Supplementary figures and images for: Case report: Advanced age at transplantation and pre-emptive treatment with dupilumab in DOCK8 deficiency
Source: Front Immunol. 2025 Jan 28;15:1507494. doi: 10.3389/fimmu.2024.1507494 (PMC11810938; doi:10.3389/fimmu.2024.1507494)

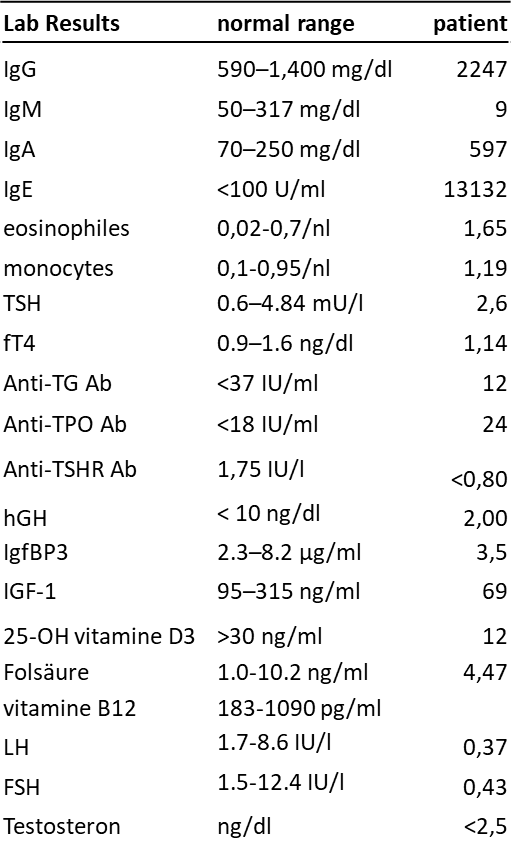

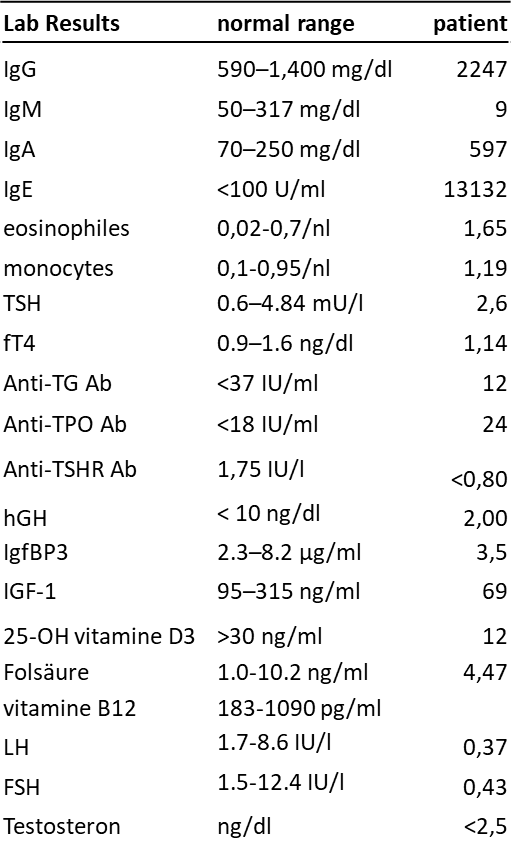

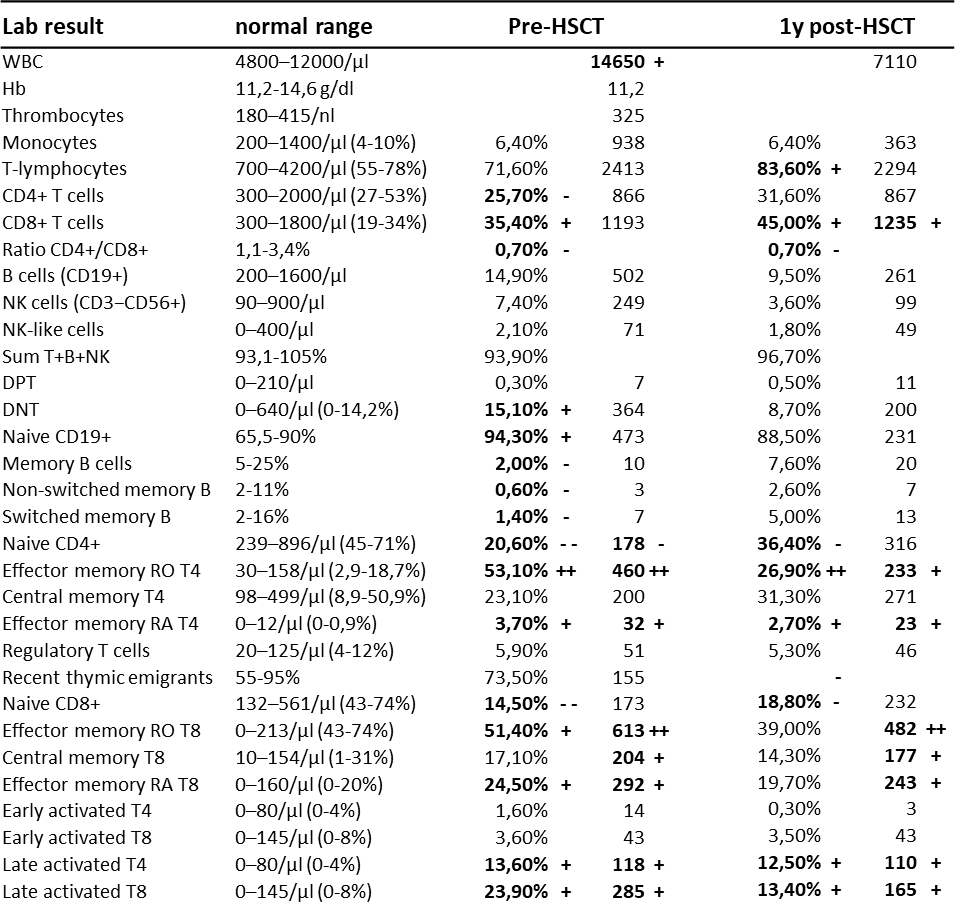
**Suppl. Table 1**

Supplement: Supplementary Table 1 — Laboratory work up of the patient with dedicator of cytokinesis 8 deficiency. WBC, white blood cell count; Hb, hemoglobin; NK, natural killer cells; DPT, double positive T cells; DNT, double-negative T cells; T4, CD4+ T helper cells; T8, CD8+ cytotoxic T cells; Ig, immunoglobulin; TSH, thyroid stimulation hormone; fT4, free thyroxine; fT3, free triiodothyronine; Ab, antibody; TG, thyroglobulin; TPO, thyroid peroxidase, TSHR, thyroid-stimulating hormone receptor; hGH, human growth hormone, IGFBP-3, insulin-like growth factor binding protein 3; IGF-1, insulin-like growth factor 1; LH, luteinizing hormone; FSH, follicle-stimulating hormone. [file Table1.docx]
